# Effect of High-Dose or Split-Dose Artesunate on Parasite Clearance in Artemisinin-Resistant Falciparum Malaria

**DOI:** 10.1093/cid/cis958

**Published:** 2012-11-21

**Authors:** Debashish Das, Rupam Tripura, Aung Pyae Phyo, Khin Maung Lwin, Joel Tarning, Sue J. Lee, Warunee Hanpithakpong, Kasia Stepniewska, Didier Menard, Pascal Ringwald, Kamolrat Silamut, Mallika Imwong, Kesinee Chotivanich, Poravuth Yi, Nicholas P. J. Day, Niklas Lindegardh, Duong Socheat, Chea Nguon, Nicholas J. White, François Nosten, Arjen M. Dondorp

**Affiliations:** 1 Mahidol Oxford Tropical Medicine Research Unit, Faculty of Tropical Medicine, Mahidol University, Bangkok; 2 Shoklo Malaria Research Unit, Mae Sot, Thailand; 3 Centre for Tropical Medicine, Nuffield Department of Clinical Medicine, University of Oxford; 4 WorldWide Antimalarial Resistance Network, Oxford, United Kingdom; 5 The National Center for Parasitology, Entomology and Malaria Control; 6 Institut Pasteur du Cambodge, Phnom Penh, Cambodia; 7 Global Malaria Programme, World Health Organization, Geneva, Switzerland

**Keywords:** artemisinins, drug resistance, *Plasmodium falciparum*, neutropenia, reticulocytopenia

## Abstract

***Background.*** The emergence of *Plasmodium falciparum* resistance to artemisinins on the Cambodian and Myanmar-Thai borders poses severe threats to malaria control. We investigated whether increasing or splitting the dose of the short-half-life drug artesunate improves parasite clearance in falciparum malaria in the 2 regions.

***Methods.*** In Pailin, western Cambodia (from 2008 to 2010), and Wang Pha, northwestern Thailand (2009–2010), patients with uncomplicated falciparum malaria were randomized to oral artesunate 6 mg/kg/d as a once-daily or twice-daily dose for 7 days, or artesunate 8 mg/kg/d as a once-daily or twice-daily dose for 3 days, followed by mefloquine. Parasite clearance and recrudescence for up to 63 days of follow-up were assessed.

***Results.*** A total of 159 patients were enrolled. Overall median (interquartile range [IQR]) parasitemia half-life (half-life) was 6.03 (4.89–7.28) hours in Pailin versus 3.42 (2.20–4.85) hours in Wang Pha (*P* = .0001). Splitting or increasing the artesunate dose did not shorten half-life in either site. Pharmacokinetic profiles of artesunate and dihydroartemisinin were similar between sites and did not correlate with half-life. Recrudescent infections occurred in 4 of 79 patients in Pailin and 5 of 80 in Wang Pha and was not different between treatment arms (*P* = .68).

***Conclusions.*** Increasing the artesunate treatment dose up to 8 mg/kg/d or splitting the dose does not improve parasite clearance in either artemisinin resistant or more sensitive infections with *P. falciparum*.

***Clinical Trials Registration.*** ISRCTN15351875.

## Abstract (precis)

New treatment strategies are needed for artemisinin-resistant falciparum malaria. This randomized trial shows that neither increasing nor splitting the standard once-daily artesunate dose reverses the markedly reduced parasite clearance rate in patients with artemisinin-resistant falciparum malaria.

**(See the Major Article by Sutanto et al, on pages 685–93** **and see the Editorial Commentary by Dondorp, on pages 694–6.)**

The emergence of partial artemisinin resistance in *Plasmodium falciparum* on the Cambodia-Thailand border and more recently on the Myanmar-Thailand border jeopardizes the renewed global efforts of control and elimination of malaria [1–4]. This poses a danger that malaria could become untreatable, as no good alternatives to artemisinin-based combination therapy (ACT) are currently available [5]. Increasing the dose of the artemisinin component in ACT might partly overcome decreased sensitivity, in case the concentration-effect relationship has merely shifted to higher concentrations in this parasite population. Increasing the dosing frequency could be another strategy to increase artemisinin efficacy.

A unique property of the artemisinin drugs is their broad-stage specificity including young ring-stage asexual parasites [6]. There is evidence that artemisinin resistance particularly involves ring-stage parasites [2, 7, 8]. This can affect the dosing frequency of the short-half-life artemisinins, which have therapeutic drug concentrations until approximately 6 hours after the dose [8]. A mathematical model predicted that twice-daily dosing of artesunate (AS) would be more effective than the conventional once-daily dose [8].

The current study explored whether increasing or splitting the once-daily AS dose increased efficacy, defined as parasite half-life [9], in patients with uncomplicated malaria in Pailin, western Cambodia, an area of artemisinin resistance, and in Wang Pha, in northwestern Thailand, where ACT has sustained efficacy since 1994 [10], although a recent decline has also been observed here [4].

## METHODS

### Study Design

In 2 open-label, randomized clinical trials, we compared different AS dosing regimens in patients with uncomplicated falciparum malaria presenting in Pailin, western Cambodia (2008–2010) and in Wang Pha, northwestern Thailand (2009–2010), areas with low and seasonal transmission. Studies were conducted according to Good Clinical Practices, and monitored independently (Family Health International). A data safety monitoring committee (DSMC) assured safety of the participants. Ethical approval was obtained from the Ministry of Health in Cambodia, the Ethics Committee of the Faculty of Tropical Medicine of Mahidol University in Thailand, the Oxford Tropical Medicine Ethical Committee, and the World Health Organization Research Ethics Review Committee.

### Patients

Patients with acute uncomplicated falciparum malaria, monoinfection, and asexual stage parasitemia between 10 000 parasites/μL and 175 000 parasites/μL assessed by microscopy were eligible provided that written informed consent was obtained from all patients or their guardian (for children). In Pailin, patients of 6 years and older were studied, whereas in Wang Pha only adult patients (≥18 years) were studied. Pregnant or lactating women were excluded as well as patients with any signs of severe infection [11]. Antimalarial treatment within 48 hours of screening, known hypersensitivity to study drugs, or splenectomy were exclusion criteria.

### Randomization and Drug Therapy

**Figure 1.**
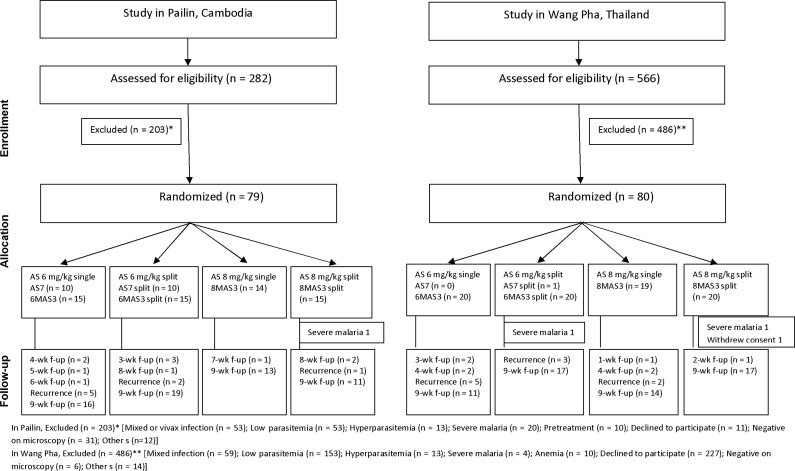
Enrollment, randomization, and follow-up of the patients in the 2 study sites. Please see the Methods section for a description of the study arms. Abbreviations: AS, artesunate; f-up, follow-up.

Patients were randomly allocated to 1 of 4 treatment arms: (1) AS alone in a dose of 6 mg/kg/d for 7 days (AS7); (2) the same total dose, but given as a split twice-daily dose (AS7_split); (3) AS in a dose of 8 mg/kg/d for 3 days, followed by mefloquine in a dose of 15 mg/kg on day 3 and 10 mg/kg on day 4 (8MAS3); (4) the same total dose, but AS given as a split twice-daily dose (8MAS3_split). Arms AS7 and AS7_split were suspended after an association with neutropenia was reported in a separate study [12]. The 7-day regimens using AS 6 mg/kg/d were then replaced by 3 days of AS 6 mg/kg/d either as single or split daily dose, followed by mefloquine 25 mg/kg divided over 2 days (Figure 1)[Fig CIS958F2].

**Figure 2. CIS958F2:**
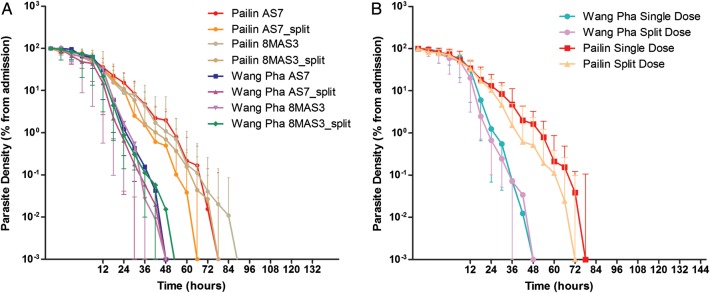
Log-linear parasite clearance curves expressed as percentage of admission parasitemia. *A*, Compares the 4 treatment arms between the 2 study sites in Pailin in western Cambodia and Wang Pha in northwestern Thailand. *B*, Compares single versus twice-daily dosing between the 2 study sites. Please see the Methods section for a description of the study arms.

Randomization was computer generated in blocks of 20. Opaque sealed envelopes were opened sequentially by a study investigator and contained unique study numbers referring to treatment allocations. AS (50-mg tablets; Guilin Pharmaceutical Co, Guangxi, China; repacked by Atlantic Laboratories Corp, Bangkok, Thailand) and mefloquine (250-mg tablets; Atlantic Laboratories Corp, Bangkok, Thailand) doses were calculated according to body weight. Mefloquine was rounded to the nearest quarter tablet and AS to the nearest half tablet in the single daily dose arm and to the nearest quarter tablet in the split-dose arm. Drug administration was directly supervised. The full dose was repeated if vomiting occurred within 30 minutes after drug administration, and half of the dose was given if within 30–60 minutes.

### Study Procedures

Patients were hospitalized for 7 days and then followed weekly until day 63 after enrollment. A detailed history and physical examination were performed and blood samples were taken for baseline biochemistry and hematology. Parasitemia was assessed at 0, 2, 4, 6, 8, and 12 hours after enrollment and then every 6 hours until 2 consecutive slides were negative. Quality checks were performed on 10% of randomly selected slides. Plasma concentrations of AS and dihydroartemisinin (DHA) were assessed at 0 (predose), 0.25, 0.5, 1, 1.5, 2, 3, 4, 5, 6, and 8 hours after the first drug administration and after the first dose of the last treatment day (day 3 or 7 depending on treatment arm). Recurrent *P. falciparum* infections were treated with quinine combined with doxycycline or clindamycin (children). Adverse events were recorded on a standard form.

### Endpoints

The primary endpoint was the parasitemia half-life (hereafter stated as “half-life”) defined by the slope of the log_e_ transformed parasite clearance curves [9]. The parasite clearance time (PCT) was defined as time of the first of 2 consecutive negative blood smears. Parasite reduction ratios (PRRs) were calculated as 100 minus the percentage reduction from baseline. PC50, PC90, and PC99 were the times needed to reduce parasitemia with 50%, 90%, and 99%, respectively, of the baseline value. Fever clearance times were defined as the time to first temperature reading <37.5°C (FCTa) and time to defervescence lasting ≥24 hours (FCTb). Efficacy outcomes at day 63, including early treatment failure, late treatment failure, and adequate clinical and parasitological response (ACPR), were classified according to standard definitions [13]. Reinfection was distinguished from recrudescent infections by standard methods using genotyping of the polymorphic genes *msp-1*, *msp-2*, and *glurp* [14].

### Pharmacokinetics

Whole blood samples were collected in prechilled fluoride-oxalate tubes and immediately centrifuged at 4°C. Plasma was stored in liquid nitrogen until analysis at the Mahidol Oxford Research Unit Pharmacology Laboratory in Bangkok. AS and DHA were extracted from plasma samples using solid phase extraction and quantified by high-performance liquid chromatography with tandem mass spectrometry [15]. Individual plasma concentration–time profiles were evaluated with noncompartmental analysis in WinNonlin version 5.0 (Pharsight Corporation). Total exposure up to the last measured concentration was calculated using the linear trapezoidal method for ascending concentrations and the logarithmic trapezoidal method for descending concentrations. The terminal elimination half-life was estimated by log-linear best-fit regression of the observed concentrations in the terminal elimination phase. Complete in vivo conversion of AS into DHA was assumed.

### Statistical Analysis

A sample size of 40 patients per arm in split versus single daily dosing of AS in artemisinin-resistant malaria was calculated to be sufficient with α = .05 and β = .80 to detect a shortening in half-life of 1.2 hours from a mean of 6.0 (SD, 1.9) hours as observed with once-daily dosing in recent studies in Pailin [2]. Normally distributed continuous data were compared using Student *t* test or analysis of variance, otherwise Mann-Whitney *U* or Kruskal-Wallis tests were used. For categorical variables, proportions were examined using χ^2^ or Fisher exact test. Rates of gametocyte carriage were calculated as total carriage time per person-weeks of follow-up. Efficacy rates were assessed by survival analysis using the Kaplan-Meier method. The rate of increase in reticulocyte counts (up to day 7) and recovery (from day 7 to day 63) was assessed using a random effects model. Analysis was performed using Stata software, version 11.2 (StataCorp).

## RESULTS

A total of 79 patients in Pailin and 80 in Wang Pha were studied between March 2008 and December 2010 (Figure 1). In total, 16 patients (10%), equally divided between sites, were lost to follow-up after discharge from hospital. Severe malaria developed shortly after admission in 1 patient in Pailin and 2 in Wang Pha. One patient in Wang Pha withdrew consent after enrollment. Baseline characteristics are shown in Table 1 and in Supplementary Table 1. Because of the difference in inclusion criteria, in Pailin 22 of 79 (28%) of patients were ≤18 years.

**Table 1.** Baseline Characteristics of Enrolled Patients According to Treatment Arm^a^ and Study Site

	Pailin, Cambodia				Wang Pha, Thailand				*P* Value
Characteristic	AS7 (n = 25)	AS7_split (n = 25)	8MAS3(n = 14)	8MAS3_split (n = 15)	AS7 (n = 20)	AS7_split(n = 21)	8MAS3(n = 19)	8MAS3_split(n = 20)	Pailin vsWang Pha
Male sex, No. (%)	23 (92)	22 (88)	11 (79)	10 (67)	19 (95)	19 (90)	18 (95)	18 (90)	.08
Age, y									.003
Mean (SD)	28 (11)	24 (9)	24 (13)	20 (8)	28 (8)	27 (6)	29 (7)	33 (12)	
Weight, kg									.18
Median	53	51	46	49	52	52	51	52	
IQR	50–56	48–54	28–53	25–54	49–55	49–57	48–54	47–56	
Temperature, °C									.0001
Mean (SD)	38.4 (1.0)	38.6 (1.0)	38.5 (0.9)	38.8 (1.0)	38.1 (1.0)	37.4 (0.6)	37.5 (0.6)	37.8 (0.8)	
Creatinine, mg/dL									.0017
Median	1.00	1.00	0.80	0.90	1.10	1.00	1.00	1.00	
IQR	0.80–1.20	0.80–1.10	0.80–1.10	0.80–1.10	0.90–1.25	0.95–1.10	0.90–1.20	0.90–1.20	
Alanine aminotransferase, U/L									.0001
Median	23	24	21	22	14	11	17	18	
IQR	20–33	19–29	16–25	19–26	8–25	8–21	9–38	10–26	
Alkaline phosphatase, U/L									.0004
Median	74	91	105	105	69	60	70	81	
IQR	66–95	83–110	61–159	69–131	64–82	57–78	66–110	69–91	
White cell count, ×10³/μL									.04
Median	5.9	6.1	6	6.3	5.9	4.5	5.5	4.9	
IQR	4.8–7.2	5.1–7.7	5.2–6.7	5.2–8.2	3.9–8.3	3.4–7.0	5.0–7.3	3.8–6.9	
Platelet count, ×10³/μL									.017
Median	106	118	119	93	96	69	83	85	
IQR	85–131	96–140	68–173	75–133	64–128	54–145	61–153	51–120	
Parasite density, parasites/μL									.18
Geometric mean	54 539	52 237	47 356	58 393	32 507	36 244	58 294	42 541	
95% CI	34 151–87 097	33 553–81 324	34 068–65 827	34 299–99 412	18 962–55 727	21 906–59 964	32 295–10 5225	27 240–66 437	
Presence of gametocytes, No. (%)	4 (16)	5 (20)	1 (7)	3 (20)	1 (5)	3 (14)	6 (32)	7 (35)	.54

Abbreviations: CI, confidence interval; IQR, interquartile range; SD, standard deviation.

^a^ Patients were randomly allocated to 1 of 4 treatment arms: (1) AS alone in a dose of 6 mg/kg/d for 7 days (AS7); (2) the same total dose, but given as a split twice-daily dose (AS7_split); (3) AS in a dose of 8 mg/kg/d for 3 days, followed by mefloquine in a dose of 15 mg/kg on day 3 and 10 mg/kg on day 4 (8MAS3); (4) the same total dose, but AS given as a split twice-daily dose (8MAS3_split). Arms AS7 and AS7_split were suspended after an association with neutropenia was reported in a separate study [12]. The 7-day regimens using AS 6 mg/kg/d were then replaced by 3 days of AS 6 mg/kg/d either as single or split daily dose, followed by mefloquine 25 mg/kg divided over 2 days.

### Parasitological Responses

**Figure 3.**
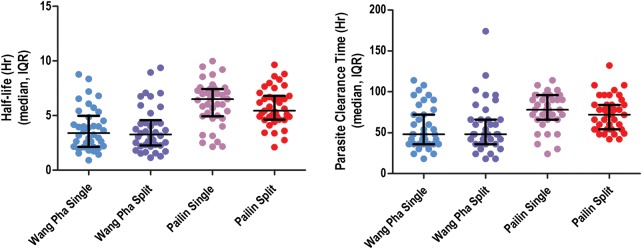
*A*, Parasitemia half-life according to study arm and study site. *B*, Parasite clearance time according to study arm and study site. Abbreviation: IQR, interquartile range.

Overall, the median half-life was 6.03 (interquartile range [IQR], 4.89–7.28) hours in Pailin versus 3.42 (IQR, 2.20–4.85) hours in Wang Pha (*P* = .0001). Within sites, there was no difference in half-life between patients receiving 6 or 8 mg/kg per day: median 6.0 (IQR, 4.9–7.5) versus 6.1 (IQR, 4.8–7.1) hours (*P* = .90) in Pailin and 3.2 (IQR, 2.2–5.1) versus 3.4 (IQR, 2.2–4.8) hours (*P* = .90) in Wang Pha (Figure 2). In Pailin half-life tended to be shorter with a split daily dose—median 6.5 (IQR, 4.9–7.4) versus 5.4 (IQR, 4.6–6.8) hours—but the difference was not statistically significant (*P* = .26, Figure 3). In Wang Pha no difference in half-life was observed with a split dose: median 3.4 (IQR, 2.2–5.0) versus 3.3 (IQR, 2.3–4.6) hours (*P* = .89).

Other measures of parasite clearance (PRR24, PRR48, PC50, PC90, PC99) confirmed the markedly slower parasite clearance in Pailin compared to Wang Pha (Table 2). The overall median PCT was 78 hours (IQR, 60–96) in Pailin versus 48 hours (IQR, 36–72) in Wang Pha (*P* = .0001) and correlated with admission parasitemia in both sites (Pailin: Kendall τ = 0.25, *P* = .001 and Wang Pha: Kendall τ = 0.40, *P* ≤ .001). Corresponding to this, at 48 hours 66 of 78 (85%) patients remained parasite positive (PPR) in Pailin versus 32 of 77 (42%) in Wang Pha (*P* < .001). At 72 hours the PPR was 41 of 78 (53%) in Pailin compared to 17 of 77 (22%) in Wang Pha (*P* < .001).

**Table 2.** Clinical and Parasitological Responses in the Study Patients^a^, According to Location

	Pailin, Cambodia				*P* Value	Wang Pha, Thailand				*P* Value	
Variable	AS7	AS7_split	8MAS3	8MAS3_split	Singlevs Split	AS7	AS7_split	8MAS3	8MAS3_split	Singlevs Split	Pailin vs Wang Pha
Patients, No.	25	25	14	14		20	20	19	18		
Parasite clearance time, h					.15					.76	.0001
Median	78	66	84	78		48	48	48	51		
IQR	66–90	54–96	72–96	54–84		36–78	36–66	36–72	42–66		
Time to 50% clearance of parasite density (PC50), h					.16					.99	.003
Median	10	9	9	8		8	6	5	7		
IQR	8–15	5–11	3–16	4–14		5–9	3–10	2–11	3–9		
Time to 90% clearance of parasite density (PC90), h					.19					.69	.0001
Median	25	18	26	22		15	13	16	14		
IQR	19–30	15–28	17–32	16–29		12–21	9–19	6–22	10–19		
Time to 99% clearance of parasite density (PC99), h					.17					.82	.0001
Median	49	37	44	45		27	24	27	25		
IQR	41–55	32–51	37–56	37–48		20–37	18–32	12–44	22–36		
Parasite reduction ratio (PRR)											
After 24 h (PRR24)					.47					.64	.0001
Median	0.85	0.9	0.91	0.89		0.99	0.99	0.99	0.99		
IQR	0.77–0.94	0.81–0.97	0.76–0.98	0.83–0.98		0.96–1.00	0.96–1.00	0.94–1.00	0.94–1.00		
After 48 h (PRR48)					.24					.89	.0001
Median	0.98	1	0.99	0.99		1	1	1	1		
IQR	0.97–1.00	0.98–1.00	0.93–1.00	0.97–1.00		1.00–1.00	1.00–1.00	1.00–1.00	1.00–1.00		
Patient parasitemic, No. (%)											
At 24 h	24/25 (96)	25/25 (100)	14/14 (100)	14/14 (100)	1	18/20 (90)	16/20 (80)	18/19 (95)	18/18 (100)	.71	.03
At 48 h	20/25 (80)	21/25 (84)	13/14 (93)	12/14 (86)	1	09/20 (45)	07/20 (35)	07/19 (37)	9/18 (50)	1	<.001
At 72 h	14/25 (56)	10/25 (40)	09/14 (64)	08/14 (57)	.26	05/20 (25)	04/20 (20)	04/19 (21)	04/18 (22)	.83	<.001
Parasitemia half life, h					.26					.89	.0001
Median	6.5	5.2	5.9	6.2		3.8	3.1	3.2	3.6		
IQR	4.9–7.4	4.9–6.7	5.0–7.1	4.6–6.8		2.4–4.8	2.0–5.1	1.9–4.8	2.3–4.3		
Fever clearance (temp <37.5°C)											
Time to first occurrence, h					.7					.5	.0005
Median	18	12	15	15		8	0	4	4		
IQR	0–24	6–24	0–24	6–30		0–18	0–12	0–18	0–12		
Time to first 24-h period, h					.23					.52	.0005
Median	36	30	33	30		18	24	24	30		
IQR	24–48	18–48	30–54	30–54		8–36	0–36	18–36	18–36		
Recrudescence, No. (%)	4 (16)	0	0	0		2 (10)	1 (5)	2 (10)	0		.68^c^
Reinfection, No. (%)	1 (4)	2 (8)	0	1 (7)		0	0	0	0		
Recurrent infection, No. (%)	5 (20)	2 (8)	0	1 (7)		5 (25)	3 (15)	2 (10)	0		.55^c^
Duration of gametocyte carriage^b^, days					.34					0.16	.72
Median	5.5	2	4	6		11	2	9.5	6		
IQR	3.5–11	2–4	4–4	1–22		11–11	1–3	2–10	1–10		

Abbreviation: IQR, interquartile range.

^a^ Patients were randomly allocated to 1 of 4 treatment arms: (1) AS alone in a dose of 6 mg/kg/d for 7 days (AS7); (2) the same total dose, but given as a split twice-daily dose (AS7_split); (3) AS in a dose of 8 mg/kg/d for 3 days, followed by mefloquine in a dose of 15 mg/kg on day 3 and 10 mg/kg on day 4 (8MAS3); (4) the same total dose, but AS given as a split twice-daily dose (8MAS3_split). Arms AS7 and AS7_split were suspended after an association with neutropenia was reported in a separate study [12]. The 7-day regimens using AS 6 mg/kg/d were then replaced by 3 days of AS 6 mg/kg/d either as single or split daily dose, followed by mefloquine 25 mg/kg divided over 2 days.

^b^ Duration of gametocyte carriage is reported only for patients who had gametocytemia at any time.

^c^ *P* value from log-rank test. The parasitemia half-life is directly proportional to the clearance rate constant describing the slope of the log-linear parasite clearance curve, a measure used in earlier publications (half-life = 0.693/parasite clearance rate).

The mean duration of patent gametocytemia per person-weeks of follow-up was 0.018 (95% confidence interval [CI], 0.014–0.022) person-weeks in Pailin and 0.029 (95% CI, 0.024–0.035) in Wang Pha (*P* = .0005). Thirteen of 79 (16%) patients in Pailin and 17 of 80 (21%) in Wang Pha had gametocytemia at some point during the study (*P* = .54). In gametocytemic patients, the median duration of gametocyte carriage was 4 (IQR, 2–7) days in Pailin and 3 (IQR, 2–10) days in Wang Pha (*P* = .72). Gametocyte carriage in patients presenting with gametocytemia was overall shorter in patients treated with a split dose of AS (median, 2.5 [IQR, 1–7] days, n = 18) compared to a single daily dose (median, 8 [IQR, 3.5–10.5] days, n = 12; *P* = .045).

### Clinical Responses

FCT was shorter in patients in Wang Pha compared to Pailin, whereas there was no difference in FCT between single and split daily-dose arms in either site (Table 2). ETF defined as parasitemia and fever ≥72 hours [13] occurred in 6 of 79 patients (8%) in Pailin, compared to 4 of 80 (5%) in Wang Pha (*P* = .53). Late parasitologic failure, defined as polymerase chain reaction (PCR)–confirmed recrudescence of *P. falciparum* infection ≥7 days after the start of treatment [13], occurred in 4 of 79 (5%) patients in Pailin after a median of 28 days (range, 28–42 days) and in 5 of 80 patients (6%) in Wang Pha, after a median of 28 days (range, 21–35 days). At day 63, the PCR unadjusted and adjusted Kaplan-Meier estimates for ACPR were 87.9% (95% CI, 78.0%–93.5%) and 93.3% (95% CI, 84.6–97.2%) in Pailin compared to 84.0% (95% CI, 73.6%–90.6%) and 90.4% (95% CI, 81.1–95.4%) in Wang Pha (P = .44 and P = .51, respectively). There was no difference in ACPR after implementation of the changes in the study arms receiving 6 mg/kg AS.

Nadir in hematocrit level was reached at day 7 in both sites, with a mean daily decrease of 0.45% per day (95% CI, .26%–.63%) in Pailin and 0.50% (95% CI, .40%–.60%) in Wang Pha. This initial drop in hematocrit level was not correlated significantly with reticulocyte counts. Reticulocyte counts on admission and days 6–7 were available from 36 patients in Pailin and 33 in Wang Pha. Compared to admission, there was a median fractional reduction after 5 days of 66.7% (IQR, 50%–82%) in Pailin and 66.7% (IQR, 0%–80%) in Wang Pha. In both sites this was not different between patients receiving 6 mg/kg/d versus 8 mg/kg/d AS (*P* = .54 and *P* = .87, respectively). Adjusted for baseline hematocrit level, recovery of reticulocyte counts after day 6 was faster in Pailin (4% per day [95% CI, 2%–5%]) compared to Wang Pha (1% [95% CI, 2%–5%]) and counts were maximal at day 14 in both sites.

The number of patients reporting minor adverse events were more frequent in Pailin (Supplementary Table 2). In Pailin, a 29-year-old man treated with a total of 24 mg/kg of AS (8MAS3) developed transient afebrile neutropenia with a nadir of 0.78 × 10^3^/μL on day 14, which normalized on day 17. In total, 2 patients in Wang Pha and 1 patient in Pailin developed severe malaria shortly after enrollment and received treatment with intravenous AS and quinine. Of these, a 30-year-old woman in Pailin unfortunately died on the fourth day of hospitalization. DSMC evaluation of the case concluded that severe malaria was the likely direct cause of death.

### Pharmacokinetics

**Figure 4.**
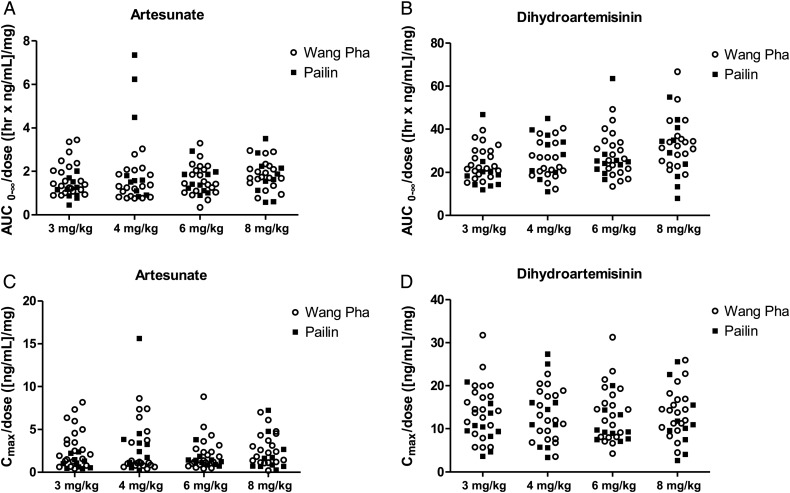
Total artesunate (*A*) and dihydroartemisinin (*B*) exposure and maximum artesunate (*C*) and dihydroartemisinin (*D*) concentrations in Pailin (filled squares) and Wang Pha (open circles) after the first dose in the different treatment arms (3 mg/kg and 4 mg/kg are twice-daily dose groups and 6 mg/kg and 8 mg/kg are once-daily dose groups). All exposure measurements are dose (mg) normalized. Abbreviations: AUC_0-∞_, predicted area under the plasma concentration time curve after the first dose from zero time to infinity; C_max_, maximum observed plasma concentration after oral administration.

The pharmacokinetic profiles of AS and DHA were comparable between sites (Table 3 and Figure 4). However, a relative small but significant higher maximum concentration was observed for both AS and DHA in Wang Pha. With increased dosing, there was a proportional increase in the maximum concentration (C_max_) and exposure (AUC_0-∞_) to both drugs (Figure 4). Compared to admission, day 7 elimination clearance values were higher and drug exposure lower for both AS (*P* = .0003) and DHA (*P* = .0001). There was no correlation between half-life and the individual exposure or maximum concentration of AS or DHA or both drugs combined. Pharmacokinetic parameters were otherwise similar to that previously reported in the same populations [2].

**Table 3.** Pharmacokinetic Parameter Estimates From the Noncompartmental Analysis of the First Dose According to Study Site

	Wang Pha (n = 79),Median (Range)	Pailin (n = 40),Median (Range)	*P* Value^a^
Body weight (kg)	51.0 (39.0–69.0)	52.0 (24.0–64.9)	.2636
Total artesunate dose (mg/kg)^a^	4.25 (2.88–8.19)	5.56 (2.86–8.33)	.2777
Artesunate			
C_max_ (ng/mL)	533 (71.1–3330)	341 (44.4–3610)	.0153
C_max_/dose ([ng/mL]/mg)	1.92 (0.203–8.83)	1.35 (0.222–15.6)	.0474
T_max_ (h)	0.500 (0.180–5.03)	0.983 (0.217–4.98)	.2296
CL/F (L/h)	645 (290–2950)	659 (136–2250)	.5699
V/F (L)	255 (65.8–3720)	358 (40.1–2430)	.0434
T_1/2_ (h)	0.279 (0.0900–2.21)	0.392 (0.125–2.73)	.0135
AUC_0-∞_ (h × ng/mL)	386 (102–1230)	375 (77.7–1470)	.3006
AUC_0-∞_/dose ([h × ng/mL]/mg)	1.55 (0.339–3.45)	1.51 (0.444–7.35)	.4925
Dihydroartemisinin			
C_max_ (ng/mL)	2430 (583–8160)	1960 (427–4050)	.0130
C_max_/dose ([ng/mL]/mg)	14.2 (3.54–31.7)	10.3 (2.60–27.3)	.0299
T_max_ (h)	1.00 (0.500–5.03)	1.48 (0.483–4.98)	.8077
CL/F (L/h)	37.1 (15.0–82.3)	41.8 (15.8–91.3)	.2442
V/F (L)	45.9 (18.0–185)	55.7 (13.1–166)	.0802
T_1/2_ (h)	0.842 (0.426–2.77)	0.836 (0.442–2.08)	.6896
AUC_0-∞_ (h × ng/mL)	4540 (1440–19700)	4680 (1260–12900)	.1630
AUC_0-∞_/dose ([h × ng/mL]/mg)	26.9 (12.2–66.7)	23.9 (7.88–63.5)	.2330

Abbreviations: AUC_0-∞_, predicted area under the plasma concentration time curve after the first dose from zero time to infinity; CL, elimination clearance; C_max_, maximum observed plasma concentration after oral administration; F oral bioavailability; T_max_, observed time to reach C_max_; T_1/2_, terminal elimination half-life; V, apparent volume of distribution.

^a^ *P* values are given using the Mann-Whitney test.

^b^ The median dose of artesunate is lower in Pailin compared to Wang Pha because of discontinuation of the 8 mg/kg/d arm in Pailin (see Figure 1).

## DISCUSSION

This study evaluated the use of an increased AS dose and an increased dosing frequency (ie, a split dose) in uncomplicated falciparum malaria with varying degrees of resistance to artemisinins. The common dose of AS is 4 mg/kg/d as a once-daily dose for 3 days as part of an ACT. This study showed that increasing the AS dose up to 8 mg/kg/d did not affect parasite clearance parameters, strongly suggesting a reduction in the maximum obtainable effect [6, 16]. There was a slight trend that splitting the AS dose in a twice-daily administration did accelerate parasite clearance in artemisinin-resistant malaria in Pailin, but this did not reach statistical significance. A mathematical model predicted a larger effect on half-life with increasing dosing frequency than observed in the current study [8]. This could be related to reduced sensitivity of not only ring-stage parasites (as assumed in the model), but also to some extent in more mature stages. Gametocyte clearance in patients presenting with gametocytemia was slightly faster with split dosing of AS, but the significance of this finding will need further study.

Further increase of the total dose of AS is limited by toxicity. A study from western Cambodia has recently shown that AS in a dose of 6 mg/kg/d for 7 days resulted in transient neutropenia with a nadir at 2 weeks after treatment. Severe neutropenia (<1.0 × 10^3^/μL) was observed in 19% of patients [12]. The findings prompted the current study to amend the dosing schedules in arm 1 and 2. In the current study, one patient treated with 8 mg/kg/d for 3 days had grade 3 transient neutropenia below 1.0 × 10^3^/μL, with no signs of concomitant infection [17]. Although transient, this dose-related neutropenia restricts increasing the currently recommended AS dose [12]. The study also showed transient reticulocytopenia that was possibly partly attributable to AS therapy, which could delay recovery of anemia. Reticulocytopenia was not dose dependent. This drug class–related effect has also been described in patients with severe malaria treated with artemisinin, artemether, and artesunate [18, 19], as well as in animal models [20].

Compared to our study in 2007–2008, there was an increase in the PPR at 72 hours (from 8% to 22%) in Wang Pha, northwestern Thailand [2]. The emergence of artemisinin resistance in northwestern Thailand was recently confirmed in a longitudinal study showing a progressive reduction in parasite clearance rates from 2001 to 2010 [4]. In the current study, the distribution of peripheral blood parasite half-life in Wang Pha did not have a unimodal shape, suggesting a separate population with prolonged parasite clearance. The emergence of artemisinin resistance on the Myanmar-Thailand border is evidently very worrying. In the current study, an unexpected 3 of 159 (1.9%) patients with uncomplicated malaria developed severe malaria after start of antimalarial treatment, an unusual sequence in susceptible infections. Whether AS-resistant falciparum malaria is indeed associated with a larger proportion of patients developing severe disease under ACT requires further study.

Recrudescence rates, which strongly depend on parasite sensitivity to the partner drug, were not different between treatment arms and sites. Despite marked slower parasite clearance in Pailin, the AS-mefloquine combination was borderline efficacious in both sites with PCR-adjusted cure rates at day 63 of 93.3% in Pailin and 90.4% in Wang Pha. In the current study with a relatively small sample size, there was no clear relationship between gametocyte carriage and asexual stage resistance to AS. A larger study is under way to address this important determinant of transmissibility [21, 22].

In agreement with previous studies, considerable interindividual variation was observed in the pharmacokinetic profiles for AS and the active metabolite DHA, but there were no major differences between sites. There was a higher maximum concentration (C_max_) of AS and DHA in Wang Pha compared to Pailin, but individual parasite clearance times were not correlated with C_max_. Total drug exposure was not different between sites. Differences in half-life between western Cambodia and northwestern Thailand can thus not be explained by differences in pharmacokinetics. There was a proportional increase in exposure with increased dosing of AS, which supports dose-independent absorption and clearance of AS and DHA. Doubling the frequency of dosing proportionately increased the duration of plasma parasiticidal drug concentrations, but this did not significantly accelerate parasite half-life. The marked decrease in total exposure for AS and DHA on day 7 compared to admission is likely to be a disease-related effect resulting from an increase in relative bioavailability during the acute phase of the disease. This has previously been reported in pregnant women on the Thai-Myanmar border [23].

In conclusion, this study suggests that slow parasite clearance in artemisinin-resistant falciparum malaria cannot be mitigated by increasing the daily dose or dose frequency of artesunate. Although ACTs were still effective in 2009–2010, this now heavily depends on efficacy of the partner drug. New classes of drugs are urgently needed because of the threat of untreatable falciparum malaria in the region.

## Supplementary Data

Supplementary materials are available at *Clinical Infectious Diseases* online (http://www.oxfordjournals.org/our_journals/cid/). Supplementary materials consist of data provided by the author that are published to benefit the reader. The posted materials are not copyedited. The contents of all supplementary data are the sole responsibility of the authors. Questions or messages regarding errors should be addressed to the author.

Supplementary Data
