# Supplementary material for: Effect of High-Dose or Split-Dose Artesunate on Parasite Clearance in Artemisinin-Resistant Falciparum Malaria
Source: Clin Infect Dis. 2012 Nov 21;56(5):e48–58. doi: 10.1093/cid/cis958 (PMC3563392; doi:10.1093/cid/cis958)
Supplement: Supplementary Data [file supp_56_5_e48__index.html]

Effect of high dose or split dose artesunate on parasite clearance in artemisinin resistant falciparum malaria — Effect of High-Dose or Split-Dose Artesunate on Parasite Clearance in Artemisinin-Resistant Falciparum Malaria — Effect of High-Dose or Split-Dose Artesunate on Parasite Clearance in Artemisinin-Resistant Falciparum Malaria — Supplementary Data 

# Effect of High-Dose or Split-Dose Artesunate on Parasite Clearance in Artemisinin-Resistant Falciparum Malaria

## Supplementary Data

Supplementary Data

**Files in this Data Supplement:**

- Supplementary Table 1 - doc file
- Supplementary Table 2 - doc file
