# Supplementary material for: Effect of High-Dose or Split-Dose Artesunate on Parasite Clearance in Artemisinin-Resistant Falciparum Malaria
Source: Clin Infect Dis. 2012 Nov 21;56(5):e48–58. doi: 10.1093/cid/cis958 (PMC3563392; doi:10.1093/cid/cis958)
Supplement: Supplementary Data [file supp_cis958_cis958supp_table1.doc]

| Characteristic | Pailin, Cambodia | |  |  | Wang Pha, Thailand | |  |  | P value |
| --- | --- | --- | --- | --- | --- | --- | --- | --- | --- |
|  | AS7 (N=25) | AS7_split (N=25) | 8MAS3 (N=14) | 8MAS3_split (N=15) | AS7 (N=20) | AS7_split (N=21) | 8MAS3 (N=19) | 8MAS3_split (N=20) | Pailin vs. Wang Pha |
| Glucose, mg/dl |  |  |  |  |  |  |  |  | 0.75 |
| Median | 117 | 104 | 121 | 122 | 110 | 113 | 121 | 118 |  |
| IQR | 108-133 | 99-117 | 110-136 | 111-127 | 99-124 | 101-118 | 110-139 | 103-139 |  |
| Bilirubin, mg/dl |  |  |  |  |  |  |  |  | 0.39 |
| Median | 1.40 | 1.40 | 1.20 | 1.80 | 1.30 | 1.46 | 1.65 | 1.71 |  |
| IQR | 1.00-1.80 | 1.00-1.60 | 1.00-1.80 | 1.00-2.40 | 0.93-1.72 | 0.86-1.81 | 1.45-2.24 | 0.76-2.46 |  |
| Aspartate aminotransferase, U/Liter |  |  |  |  |  |  |  |  | 0.21 |
| Median | 32 | 34 | 30 | 33 | 32 | 28 | 31 | 32 |  |
| IQR | 25-47 | 24-39 | 26-42 | 27-46 | 25-36 | 24-36 | 23-39 | 24-37 |  |
| Hematocrit, % |  |  |  |  |  |  |  |  | 0.25 |
| Mean (SD) | 38 (4) | 39 (4) | 36 (7) | 40 (7) | 40 (4) | 39 (6) | 40 (6) | 39 (5) |  |
| Hemoglobin, g/dl |  |  |  |  |  |  |  |  | 0.46 |
| Mean (SD) | 12.6 (1.5) | 12.8 (1.4) | 11.9 (2.2) | 13.3 (2.5) | 13.1 (1.5) | 12.7 (2.3) | 12.9 (2.1) | 12.8 (2.3) |  |
| Neutrophil, % |  |  |  |  |  |  |  |  | 0.33 |
| Median | 74 | 70 | 74 | 73 | 72 | 62 | 73 | 73 |  |
| IQR | 65-79 | 52-79 | 59-81 | 62-86 | 62-79 | 54-75 | 54-82 | 68-77 |  |

Table S1: Numbers are mean (SD) or median (IQR) unless otherwise stated.
