# Supplementary material for: Effect of High-Dose or Split-Dose Artesunate on Parasite Clearance in Artemisinin-Resistant Falciparum Malaria
Source: Clin Infect Dis. 2012 Nov 21;56(5):e48–58. doi: 10.1093/cid/cis958 (PMC3563392; doi:10.1093/cid/cis958)
Supplement: Supplementary Data [file supp_cis958_cis958supp_table2.doc]

|  | Pailin |  | Wang Pha |  | P |
| --- | --- | --- | --- | --- | --- |
| Adverse events | AS single (n=39) | AS split (n=39) | AS single (n=39) | AS split (n=40) | Pailin vs Wang Pha |
| Dizziness | 30 | 32 | 17 | 20 | <0.001 |
| Headache | 36 | 38 | 11 | 7 | <0.001 |
| Nausea | 30 | 29 | 3 | 2 | <0.001 |
| Anorexia | 25 | 29 | 3 | 4 | <0.001 |
| Vomiting | 10 | 12 | 6 | 5 | 0.03 |
| Diarrhoea | 5 | 1 | 3 | 3 | 0.98 |
| Itching | 2 | 1 | 2 | 2 | 1.0 |
| Rash | 1 | 1 | 1 | 0 | 0.62 |
| Urticaria | 0 | 0 | 0 | 0 | - |
| Abdominal pain | 18 | 18 | 2 | 3 | <0.001 |
| Joint pain | 17 | 15 | 10 | 11 | 0.06 |
| Muscle pain | 15 | 15 | 7 | 9 | 0.01 |
| Palpitation | 5 | 4 | 1 | 5 | 0.4 |
| Sleep problem | 14 | 16 | 2 | 4 | <0.001 |
| Confusion | 0 | 1 | 0 | 0 | 0.49 |
| Hearing loss | 5 | 3 | 0 | 0 | 0.003 |
| Numbness | 0 | 1 | 1 | 1 | 1.0 |
| Blurred vision | 3 | 2 | 0 | 1 | 0.12 |
| Fatigue | 26 | 32 | 6 | 1 | <0.001 |
| Other* | 7 | 2 | 2 | 4 | 0.4 |

Table S2: Adverse events: Number of patients experiencing symptoms at least once during antimalarial treatment. * ‘Others’ include cough, sore throat, chest pain, and ‘red eye’
